# Biphasic Euchromatin-to-Heterochromatin Transition on the KSHV Genome Following *De Novo* Infection

**DOI:** 10.1371/journal.ppat.1003813

**Published:** 2013-12-19

**Authors:** Zsolt Toth, Kevin Brulois, Hye-Ra Lee, Yoshihiro Izumiya, Clifford Tepper, Hsing-Jien Kung, Jae U. Jung

**Affiliations:** 1 Department of Molecular Microbiology and Immunology, Keck School of Medicine, University of Southern California, Los Angeles, California, United States of America; 2 Department of Dermatology, University of California Davis School of Medicine, University of California Davis Comprehensive Cancer Center, Sacramento, California, United States of America; 3 Department of Biological Chemistry and Molecular Medicine, University of California Davis School of Medicine, University of California Davis Comprehensive Cancer Center, Sacramento, California, United States of America; University of North Carolina at Chapel Hill, United States of America

## Abstract

The establishment of latency is an essential step for the life-long persistent infection and pathogenesis of Kaposi's sarcoma-associated herpesvirus (KSHV). While the KSHV genome is chromatin-free in the virions, the viral DNA in latently infected cells has a chromatin structure with activating and repressive histone modifications that promote latent gene expression but suppress lytic gene expression. Here, we report a comprehensive epigenetic study of the recruitment of chromatin regulatory factors onto the KSHV genome during the pre-latency phase of KSHV infection. This demonstrates that the KSHV genome undergoes a biphasic chromatinization following *de novo* infection. Initially, a transcriptionally active chromatin (euchromatin), characterized by high levels of the H3K4me3 and acetylated H3K27 (H3K27ac) activating histone marks, was deposited on the viral episome and accompanied by the transient induction of a limited number of lytic genes. Interestingly, temporary expression of the RTA protein facilitated the increase of H3K4me3 and H3K27ac occupancy on the KSHV episome during *de novo* infection. Between 24–72 hours post-infection, as the levels of these activating histone marks declined on the KSHV genome, the levels of the repressive H3K27me3 and H2AK119ub histone marks increased concomitantly with the decline of lytic gene expression. Importantly, this transition to heterochromatin was dependent on both Polycomb Repressive Complex 1 and 2. In contrast, upon infection of human gingiva-derived epithelial cells, the KSHV genome underwent a transcription-active euchromatinization, resulting in efficient lytic gene expression. Our data demonstrate that the KSHV genome undergoes a temporally-ordered biphasic euchromatin-to-heterochromatin transition in endothelial cells, leading to latent infection, whereas KSHV preferentially adopts a transcriptionally active euchromatin in oral epithelial cells, resulting in lytic gene expression. Our results suggest that the differential epigenetic modification of the KSHV genome in distinct cell types is a potential determining factor for latent infection versus lytic replication of KSHV.

## Author Summary

Although the KSHV genome is linear and chromatin-free in the virions, it circularizes and adopts a repressive chromatin structure in latently infected cells, inhibiting the majority of viral gene expression. In this study, we investigate the epigenetic regulatory mechanism of the pre-latency phase of KSHV infection. We found that upon *de novo* infection, the KSHV genome undergoes distinct chromatin states in a temporally ordered manner prior to the establishment of latency. Initially, the KSHV genome carried a transcriptionally permissive chromatin structure to allow expression of a subset of viral lytic genes. Subsequently, cellular Polycomb Repressive Complex 1 (PRC1) and PRC2 were recruited to the KSHV genome, resulting in the deposition of repressive histone marks onto the viral chromatin and the accumulation of heterochromatin structures, both of which were critical for the establishment of viral latency. In contrast to the biphasic chromatinization and genome-wide inhibition of lytic genes observed in *de novo*-infected SLK and TIME cells, KSHV adopts a transcriptionally permissive chromatin form in human gingiva-derived epithelial cells, resulting in prolonged and robust lytic gene expression. Thus, our results suggest that the differential epigenetic modification of the KSHV genome in distinct cell types is a potential determining factor for latent infection versus lytic replication of KSHV.

## Introduction

Kaposi's sarcoma-associated herpesvirus (KSHV, Human herpesvirus 8 or HHV-8) is one of the seven currently known human tumor viruses and is associated with the pathogenesis of the multifocal, angiogenic and inflammatory cancer called Kaposi's sarcoma (KS) and certain B cell-originated neoplasias, including primary effusion lymphoma (PEL) and multicentric Castleman's disease (MCD) [1], [2]. KSHV results in persistent infection in immunocompetent humans by establishing latency in CD19^+^ B cells [3]. The establishment of latency is the most fundamental immune evasion strategy of KSHV, as the severely limited viral gene expression characteristic of latently infected cells allows the virus to escape detection by the host immune system. However, immune suppression along with other environmental and physiological factors can trigger the reactivation of KSHV from latency, leading to the temporally ordered expression of viral genes and release of infectious virus [4], [5].

In KSHV-associated tumors, the majority of tumor cells harbor KSHV in the latent phase and virus production is restricted to a small population, indicating that it is the latently infected cells that play a critical role in the development of KSHV-associated cancers [6]. Indeed, the latent proteins of KSHV have several important roles, such as the promotion of malignant transformation by facilitating the proliferation and survival of infected cells, as well as the maintenance of the KSHV genome in dividing cells [7]. During latency, the KSHV genome exists as a circular episome in the nucleus and adopts a nucleosome structure similar to the bulk chromatinized cellular genome [8], [9]. In this latent phase, the latent genes of KSHV are continuously expressed, while the lytic genes are repressed. Since chromatinization limits the access of transcription factors to the promoter regions of viral genes, modification of the viral chromatin plays an essential role in the control of viral gene expression. Based on the different combinations of activating (acetylated H3K9/K14 or acH3 and H3K4me3) and repressive (H3K9me3 and H3K27me3) histone modifications that can be found on the chromatin of the KSHV genome during latency, we have previously shown that the chromatin of the viral episome is organized into distinct domains [10], [11], [12].

One of the major cellular transcription repressors is the Polycomb Repressive Complex 2 (PRC2), which is composed of three core subunits (EZH2, EED and SUZ12) and can interact with other transcription repressors, such as histone deacetylases (HDACs), H3K4me3 demethylases and DNA methyltransferases [13]. EZH2 catalyzes the trimethylation of histone H3K27, which is one of the hallmarks of PRC2 function. We have previously shown that both EZH2 and H3K27me3 are highly enriched on lytic gene-coding regions of the KSHV genome during latency and their association with the viral DNA decreases upon reactivation, indicating that PRC2 plays a critical role in the repression of lytic gene expression [11]. PRC2 often co-operates with another polycomb group protein (PcG) complex called PRC1 in the inhibition of cellular genes involved in cell proliferation, differentiation and development. The enzymatic subunits of PRC1 are the RING1A/B E3 ubiquitin ligases, which mono-ubiquitinate lysine 119 of H2A (H2AK119ub). This modification is thought to play a role in chromatin condensation and transcription repression [14]. It has been shown that the PRC1 can be recruited to its target promoters via the CBX protein, a subunit of PRC1 that binds to H3K27me3, suggesting that PRC1 is recruited to its target loci in a PRC2-dependent manner. However, there are at least six different PRC1 complexes, and the RING1 and YY1 binding protein (RYBP) DNA-binding factor-containing PRC1 can also be recruited to several PcG target sites and repress cellular genes independently of PRC2 [15], [16]. Whether PRC1 is also recruited to the KSHV genome during latency and contributes to the repression of lytic genes has not yet been addressed.

Establishment of latency is an essential step for herpesvirus persistent infection. KSHV can infect a variety of cell types where it establishes latency in the majority of cases, suggesting that lytic gene expression is constantly inhibited and only latent gene expression is permitted [6]. Although the KSHV genome exists in a linear and histone-free form in the viral capsid, upon infection, it becomes a closed circular episome that subsequently associates with cellular histones and persists as a non-integrated minichromosome in the nucleus of infected cells [9], [10]. Interestingly, it has been shown that some lytic genes possessing immunomodulatory or anti-apoptotic functions are temporarily expressed after infection [17]. These observations prompted us to hypothesize that the KSHV genome undergoes a dynamic transition from an active to a repressive chromatin state following *de novo* infection, which allows transient lytic gene expression prior to the establishment of latency.

To investigate the molecular details of the “pre-latency” phase of KSHV infection, we analyzed the recruitment of chromatin regulatory factors onto the KSHV genome following *de novo* infection. Based on our results, we propose that the KSHV genome undergoes a biphasic chromatinization after *de novo* infection. Initially, a transcriptionally active euchromatin, characterized by high levels of H3K4me3 and acetylated H3K27 (H3K27ac), is deposited on the viral episome and is later switched to the PcG protein-regulated heterochromatin. We show that both PRC2 and PRC1 are involved in the inhibition of lytic gene expression following *de novo* infection. Furthermore, while the KSHV genome undergoes a temporally ordered euchromatin-to-heterochromatin transition in infected endothelial cells, KSHV adopts a transcriptionally active euchromatin form in oral epithelial cells, resulting in lytic gene expression. Thus, we hypothesize that the deposition of differential epigenetic modifications on the KSHV genome in distinct cell types potentially determines whether KSHV infection results in latent or lytic replication.

## Results

### Gradual chromatinization of the KSHV genome following *de novo* infection

We investigated how the chromatin of the latent KSHV genome formed on the initially histone-free KSHV genome following *de novo* infection. We primarily used SLK cells as a model system for the *de novo* KSHV infection experiments for the following reasons: (i) SLK cells are highly susceptible to KSHV infection leading to the establishment of latency, which is thought to be the default pathway of natural KSHV infection, (ii) SLK cells support lytic replication upon treatment with chemical inducers or the overexpression of the replication and transcription activator protein (RTA) of KSHV, and (iii) the histone modification pattern of KSHV chromatin in latently infected SLK cells significantly resembles that in PEL cells [12], [18]. Furthermore, we used the recombinant KSHV BAC16 throughout the study that constitutively expresses GFP [19]. FACS and immunofluorescence analysis indicated that nearly 100% of SLK cells were GPF-positive at 16–24 hours post-infection (hpi), showing the efficiency of infecting SLK cells with KSHV (data not shown and Figure S1).

**Figure 1:**
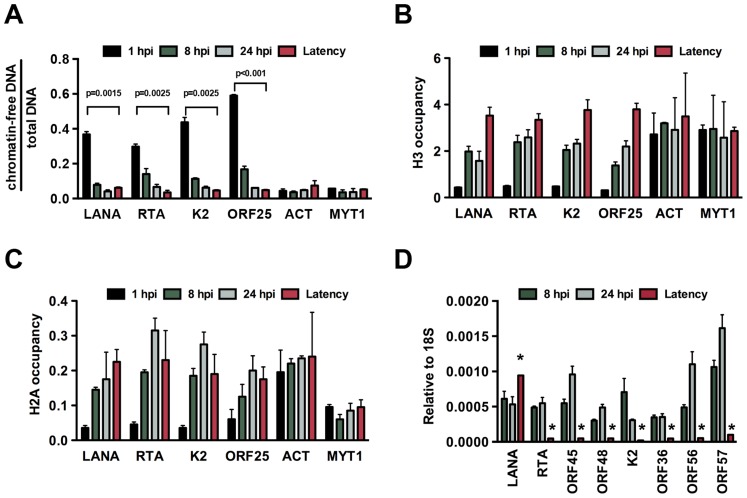
*De novo* chromatinization of the KSHV genome following infection. (**A**) FAIRE assay showing the degree of chromatinization of the indicated viral and cellular promoters in SLK cells infected for 1, 8 or 24 hours or in latently infected SLK cells. (**B**) and (**C**) The enrichment of histones H3 and H2A was calculated as ChIP/input (% Input or occupancy) at the indicated viral and cellular promoters following *de novo* infection at 1, 8 and 24 hpi and in latently infected SLK cells. (**D**) Real time RT-PCR analysis was performed to determine the expression of the indicated viral genes in SLK cells infected by KSHV for 8 and 24 hours and in latently infected SLK cells. Expression levels of viral genes are shown relative to those of 18S. A 2-tailed student's t-test was performed between 8 hpi and latency for each tested lytic gene and all p values were less than 0.05.

In order to investigate the chromatin assembly on the viral genome following *de novo* infection, we performed FAIRE (Formaldehyde-assisted isolation of regulatory elements) analysis, which technique had been used to identify nucleosome depleted regions in the KSHV genome [20] and histone occupancy measurements on several KSHV promoters in SLK cells at 1, 8 and 24 hpi (Figure 1). Latently infected SLK cells that were maintained for more than 6 months after initial KSHV infection were used as a reference point to represent fully chromatinized viral episomes. The selected viral promoters represent the gene regulatory regions of the four kinetic classes of KSHV genes: latency (LANA), immediate early (IE, RTA), early (E, K2) and late (L, ORF25). The promoters of actin (ACT) and myelin transcription factor 1 (MYT1) served as cellular controls. The FAIRE assay is used for the separation of chromatin-free DNA fragments from the chromatin-associated ones based on their differential retention in the aqueous phase during phenol-chloroform extraction. Chromatin-free DNA fragments in the aqueous phase are subsequently purified and measured by real-time quantitative PCR analysis. Figure 1A shows the relative amounts of chromatin-free viral and cellular promoter DNA fragments purified from infected cells at different time-points. These results revealed that a significant proportion of the viral promoters were initially chromatin-free, but they rapidly underwent chromatinization and the degree of their chromatin association ultimately became similar to that of the cellular genes (Figure 1). Accordingly, the H3 and H2A histone occupancy of the KSHV genome gradually increased shortly after *de novo* infection, indicating the assembly of the KSHV genome into a nucleosome structure, which ultimately resulted in comparable levels of histone H3 and even higher enrichment of H2A on the viral genome relative to the cellular genome (Figures 1B and C). Furthermore, in accordance with previous findings [17], we detected transient expression of lytic genes upon *de novo* infection and found higher levels of lytic gene expression at 24 hpi compared to latently infected cells (Figures 1D and S1). These data indicate that the KSHV genome undergoes rapid chromatinization following infection, suggesting that the initial burst of lytic gene transcription likely originates from transcriptionally permissive chromatin rather than naked DNA.

### Temporally ordered deposition of activating and repressive histone modifications on the KSHV genome during *de novo* infection

**Figure 2:**
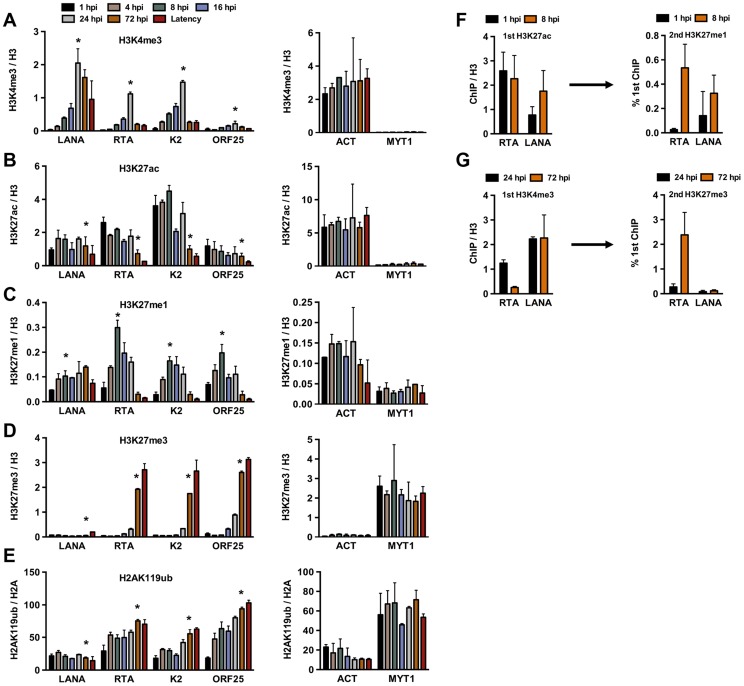
Analysis of the deposition of histone modifications on KSHV promoters during *de novo* infection using time-course ChIP assays. ChIPs were performed using latently infected SLK cells or SLK cells following *de novo* infection for the indicated hours post infection (hpi) and the enrichment of histone modifications was measured by qPCR using primers specific for the indicated viral and cellular promoters. ChIPs for each histone modification were normalized for the amount of the relevant histones at each promoter. T-test was applied to compare the values between the indicated time points (*). (**A**) H3K4me3 ChIP (for LANA, RTA and K2 p<0.05, for ORF25 p = 0.1 between 1 and 24 hpi). (**B**) H3K27ac ChIP (for LANA p = 0.6, RTA and K2 p<0.05, for ORF25 p = 0.18 between 1 and 72 hpi). (**C**) H3K27me1 ChIP (for LANA p = 0.063, for RTA, K2 and ORF25 p<0.05 between 1 and 8 hpi). (**D**) H3K27me3 ChIP (for LANA p = 0.33, for RTA, K2 and ORF25 p<0.005 between 1 and 72 hpi). (**E**) H2AK119ub ChIP (for LANA p = 0.3, for RTA, K2 and ORF25 p<0.02 between 1 and 72 hpi). (**F**) Sequential deposition of H3K27ac and H3K27me1 on viral promoters during infection was confirmed by sequential ChIP assays. The first ChIP was performed with H3K27ac-specific antibody at 1 hpi and 8 hpi, followed by elution of the immunoprecipitated DNA, The eluted DNA was used as the input for a second ChIP performed with H3K27me1 antibody. (**G**) Sequential deposition and colocalization of H3K4me3 and H3K27me3 on the RTA promoter following *de novo* infection were confirmed by sequential ChIP assays. The first ChIP was performed with H3K4me3-specific antibody at 24 hpi and 72 hpi followed by the second ChIP for H3K27me3 using the eluted first ChIPs as the input.

In order to investigate whether the deposition of activating and repressive histone marks on the KSHV genome occurs in a spatially and temporally regulated manner following *de novo* infection, we performed the ChIP assay on the KSHV genome at 1, 4, 8, 16, 24 and 72 hpi in SLK cells (Figure 2). The chromatin of the latent KSHV genome was used as a reference, as previously described [12]. In addition, the histone modification ChIPs were normalized for the total amount of relevant histone at a given genomic region. The transcriptionally active promoter of cellular actin (ACT) gene and the transcriptionally silenced promoter of cellular Polycomb-targeted MYT1 gene were used as controls to show the comparable efficacy of histone mark ChIPs at each time point throughout the experiments. This time course ChIP assay revealed a temporally ordered deposition of activating and repressive histone modifications on the KSHV genome following *de novo* infection. Specifically, the activating H3K4me3 histone mark gradually increased on the latent (LANA), IE (RTA) and E (K2) promoters, peaking at 24 hpi and then declining by 72 hpi, while the level of H3K4me3 on the L (ORF25) promoter was considerably lower compared to those of other promoters at all of the time points analyzed (Figure 2A). The H3K27 can be either acetylated (H3K27ac), characteristic for transcriptionally active genes, or mono- (H3K27me1), di- or trimethylated, characteristic for transcriptionally inactive genes. H3K27ac was detected on the latent, IE and E promoters as early as 1 hpi, declined by 72 hpi and remained low on the IE and E promoters during latency (Figure 2B). H3K27me1 significantly increased on the lytic promoters, peaked at 8 hpi, and declined thereafter, while H3K27me3 started to increase on lytic promoters at 24 hpi, and reached a level comparable to that seen on the latent genome by 72 hpi (Figure 2C and D). As shown with repressed cellular genes where the PRC2-mediated H3K27me3 often coexists with the PRC1-mediated H2AK119ub histone modification, the H2AK119ub was also enriched on the lytic promoters in conjunction with H3K27me3 (Figure 2E). The levels of these histone modifications on the cellular promoters (ACT and MYT1) remained similar in the course of *de novo* infection, suggesting that the histone modification changes specifically occur on the viral genome (Figure 2A–E). In addition, we showed that the temporally ordered deposition of activating and repressive histone marks on the KSHV genome was not restricted to the infected SLK cells, as it was also detected in *de novo*-infected TIME cells (Figure S2).

To determine whether the temporally ordered deposition of histone marks occurs on the same KSHV episome, sequential ChIP assays were applied. In the first set of experiments, H3K27ac ChIP was performed initially at 1 hpi and 8 hpi, followed by the elution of the immunoprecipitated chromatin for use in a second ChIP with anti-H3K27me1 antibody (Figure 2F). ChIP DNAs were quantified by qPCR using specific primers for the promoters of RTA and LANA genes. This showed that H3K27me1 coexisted with H3K27ac on the same viral genome at 8 hpi. In the second set of sequential ChIPs, H3K4me3 ChIP was performed at 24 and 72 hpi, followed by second ChIPs with anti-H3K27me3 antibody (Figure 2G). These results showed that the RTA promoter carried a high level of H3K4me3 and a low level of H3K27me3 at 24 hpi, whereas a large amount of H3K27me3 was deposited onto the RTA promoter associated with a low level of H3K4me3 at 72 hpi (Figure 2G). In contrast, the H3K4me3-enriched LANA promoter remained relatively H3K27me3-free (Figure 2G). This finding is in accordance with the previous findings that the RTA promoter possesses a bivalent chromatin, evidenced by the presence of both activating and repressive histone modifications on this promoter during latency [11], [12]. Taken together, our results show that KSHV undergoes different chromatin states upon *de novo* infection before it adopts the H3K27me3/H2AK119ub-enriched heterochromatin characteristic of latency. Specifically, the viral genome has a transcriptionally permissive chromatin immediately after infection, which is then switched to transcriptionally repressive chromatin. In addition, the switch from active to repressive chromatin is concurrent with the inhibition of lytic gene expression.

### Dynamic and global changes of histone modifications on the KSHV genome following *de novo* infection

**Figure 3:**
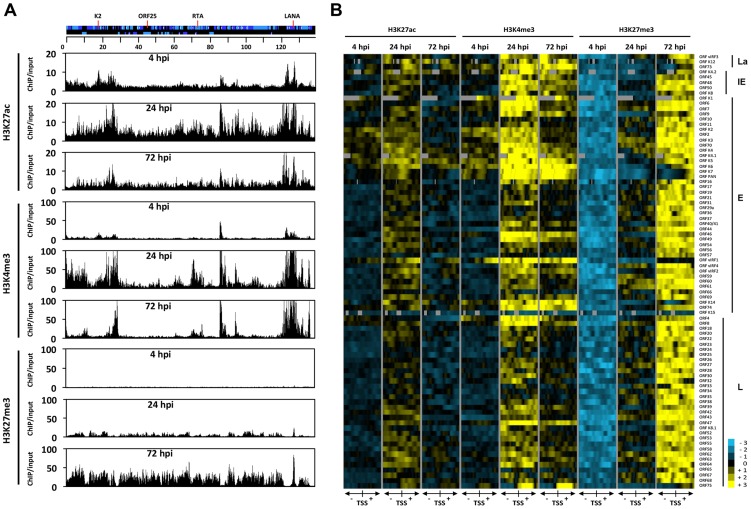
Genome-wide view of the deposition of histone modifications on the KSHV genome following *de novo* infection. (**A**) ChIP-on-chip was performed for H3K27ac, H3K4me3 and H3K27me3 at 4, 24 and 72 hpi using SLK infected cells. The alternating dark and light blue squares represent viral ORFs. (**B**) Heat map representation of changes in histone modifications at the gene regulatory regions of KSHV genes grouped by expression class (latent, La, immediate-early, IE, early, E, late, L). The rows display the relative abundance of the indicated histone modification within the −1 kb to +1 kb genomic regions flanking the translational start site (TSS) of each viral gene. The blue and yellow colors denote lower-than-average and higher-than-average enrichment, respectively, whereas gray represents missing values for enrichment due to lack of probes in those genomic regions.

In order to obtain a genome-wide and comprehensive view of the changes in viral chromatin that occur following *de novo* infection, a series of ChIP-on-chip experiments was performed. We mapped the genome-wide deposition of the activating histone marks, H3K27ac and H3K4m3, and the repressive histone mark, H3K27me3, on the KSHV genome in SLK cells at 4, 24 and 72 hpi (Figure 3A). For this, we used our KSHV-specific 15-bp tiling microarray, which contains 60 nucleotide-long oligos covering the entire KSHV genome and enables high resolution mapping of histone marks on the viral genome [11]. Based on the genome-wide ChIP data, we generated a heat map that offered a close-up view of the chromatin structure of the regulatory regions of KSHV genes and quantified the changes that occurred in these regions following *de novo* infection (Figure 3B). For this, we plotted the signal intensities of probes derived from the ChIP-on-chip analysis at 1 kb upstream and 1 kb downstream of the translational start site (TSS), as previously described [11]. The rationale of this strategy is based on the considerations that (i) due to the compact structure of the KSHV genome, the promoters are generally closely localized upstream of the TSS and (ii) the distinct modification of histones in the 5′ region of the gene bodies usually plays a role in the regulation of gene expression.

ChIP-on-chip experiments revealed highly dynamic and global changes in the posttranslational modifications of the viral chromatin following *de novo* infection (Figure 3). In agreement with our initial gene-specific ChIP experiments, we found that while activating histone marks were detected on the viral chromatin as early as 4 hpi, the repressive H3K27me3 histone mark was completely absent at an initial stage. Furthermore, our analyses revealed that the genome-wide enrichment of the activating histone marks H3K27ac and H3K4me3 highly correlated with each other on the KSHV genome throughout infection (Table S2 and Figure 3). The high Pearson correlation of the enrichment of H3K27me3 on the KSHV genome between 24 hpi and 72 hpi indicated that EZH2 was targeted to those genomic regions as early as 24 hpi, where the H3K27me3-rich chromatin domain would ultimately be established at 72 hpi (Table S2). In addition, our analysis indicated that the histone modification changes on the viral genome were highly gene specific: (i) The latency locus, which encodes constitutively expressing genes, such as ORF73/LANA, adopted activating histone modifications H3K27ac and H3K4m3 during *de novo* infection. (ii) While the activating histone marks were initially enriched at a few IE (K4.2, ORF48 and ORF50/RTA and E lytic genes (e.g. K2, K3, K4, K5, K6 and vIRF1) at 4 hpi, this enrichment expanded to nearly all lytic genes at 24 hpi, and subsequently declined concomitantly with the increased enrichment of the repressive histone mark H3K27me3 occurring between 24 and 72 hpi. (iii) Interestingly, the gene regulatory regions of IE and E genes (e.g. K5, K6, K7, ORF74 and vIRF1) were largely devoid of H3K27me3 at 24 hpi, where both H3K4me3 and H3K27ac were highly enriched. (iv) While the enrichment of H3K27ac was dramatically reduced and restricted to a few genes (e.g. ORF73, K4.2, K4, K5, K6 and vIRF1) by 72 hpi, a high level of H3K4me3 remains at several genomic regions (e.g. 15–30 kb, 70–90 kb) mainly encoding IE and E lytic genes. (v) Genomic regions encoding a large number of late genes (e.g. 30–60 kb and 95–115 kb) showed low levels of activating histone modifications at 72 hpi and were enriched with the repressive histone mark, H3K27me3. These results indicate that the majority of the KSHV genome undergoes a biphasic euchromatin-to-heterochromatin transition after *de novo* infection.

### RTA facilitates the deposition of activating histone modifications on the viral genome following *de novo* infection

**Figure 4:**
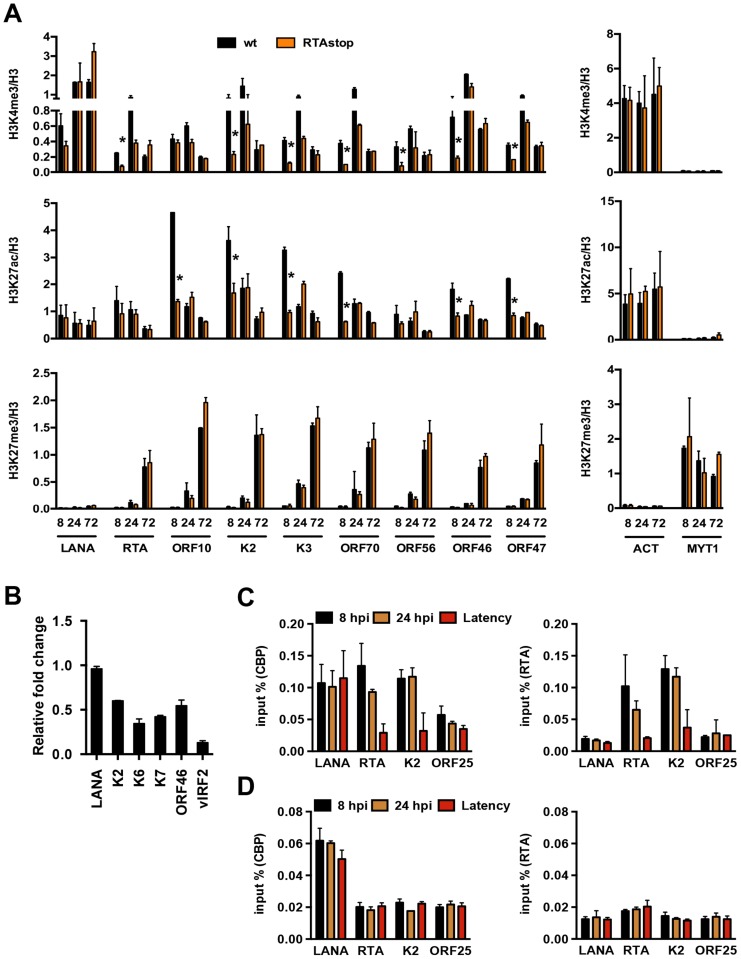
RTA is involved in the deposition of activating histone marks on the KSHV genome following *de novo* infection. (**A**) Comparative ChIP analysis of H3K4me3, H3K27ac and H3K27me3 at the indicated viral promoters of wild type (wt) or RTA knockout (RTAstop) KSHV-infected SLK cells at 8, 24 and 72 hpi. The promoters of the cellular actin (ACT) and MYT1 genes were used as controls. ChIPs were normalized for the amount of histone H3 at each promoter. The asterisk denotes p<0.05 between wt and RTAstop at 8 hpi. (**B**) RT-qPCR analysis of viral gene expression in RTAstop KSHV-infected SLK cells at 24 hpi. The expression of the indicated viral genes was calculated relative to wt KSHV-infected cells. (**C**) ChIP experiments showing the binding of RTA and CBP on the RTA-responsive RTA and K2 promoters as well as the LANA promoter in latently-infected cells and naïve cells infected for 8 or 24 hours by wild type KSHV. ORF25 promoter was used as a negative control. (**D**) RTA and CBP binding on viral promoters in RTAstop KSHV-infected SLK cells.

Previous studies have shown that a subset of lytic genes, including RTA, is temporarily expressed following *de novo* infection [17]. To address whether the transient expression of RTA plays a role in the regulation of chromatinization of the KSHV genome during *de novo* infection, we infected SLK cells with either wild type (wt) or RTA knockout (RTAstop) KSHV [21] and performed ChIP assays for H3K4me3, H3K27ac and H3K27me3 at 8, 24 and 72 hpi. Interestingly, the levels of both H3K4me3 and H3K27ac were significantly lower on several lytic promoters at 8 hpi in RTAstop virus-infected cells relative to WT virus-infected cells, while the deposition of H3K27me3 was similar in both cells (Figure 4A). In contrast, the levels of these histone modifications were comparable on the LANA promoter and the cellular ACT and MYT1 promoters in WT virus- vs. RTAstop virus-infected cells (Figure 4A). Consequently, the transient induction of lytic genes (K2, K6, K7, ORF46 and vIRF2) was lower in RTAstop virus-infected cells compared to WT virus-infected cells (Figure 4B). Since RTA has been shown to bind to its responsive viral promoters (e.g. RTA and K2) and recruits CBP, the histone acetyltransferase of H3K27ac [22], we also performed ChIP assays for RTA and CBP and found that both RTA and CBP bound to the RTA and K2 promoters following *de novo* infection, but not during latency, whereas their binding was not observed on the ORF25 promoter, which lacks RTA responsive elements (Figure 4C). Furthermore, while CBP-binding to the RTA and K2 promoters was abolished in RTAstop KSHV-infected cells, it was still detected on the LANA promoter possessing the H3K27ac mark (Figure 4D). These data indicate that CBP can be recruited to the KSHV genome by two distinct mechanisms. The KSHV RTA recruits CBP to its responsive lytic promoters following *de novo* infection and thereby facilitates the deposition of activating histone marks on the KSHV genome. Alternatively, CBP can be recruited to the KSHV genome independently of RTA.

### Overexpression of RTA during *de novo* infection results in constitutive lytic replication of infected KSHV

While KSHV RTA is sufficient to induce robust expression of lytic genes and completion of a full cycle of lytic replication [11], [23], its temporal expression during *de novo* infection leads only to the transient expressions of a few lytic genes prior to the establishment of latency [17]. Therefore, we asked whether the continuous expression of RTA during *de novo* infection affected the chromatinization of the viral genome. Doxycycline (Dox)-inducible RTA-expressing iSLK cells [18] were pre-treated with Dox for 8 hours, followed by KSHV infection for 24 and 72 hours. Indeed, continuous RTA expression during *de novo* infection not only led to the induction of lytic gene expressions and full-scale viral replication (Figure S3A, B and C), but also reduced chromatinization of the replicating KSHV genome, evidenced by the reduction of histones H3 and H2A occupancy on the KSHV promoters (Figure S3D). These data indicate that, unlike overexpression of RTA, the transient expression of RTA during *de novo* infection may not be sufficient to induce the full lytic gene expression program.

### PRC2 and PRC1 complexes bind to the KSHV genome and promote the inhibition of lytic genes following *de novo* infection

**Figure 5:**
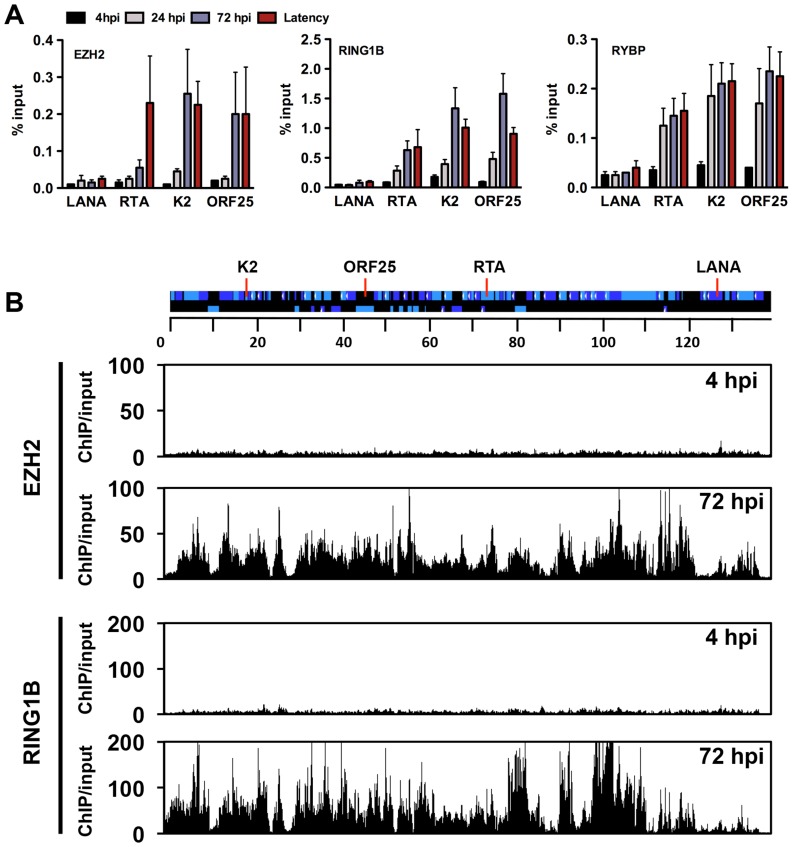
Recruitment of components of the PRC2 and PRC1 complexes onto the KSHV genome during *de novo* infection. (**A**) Time-course ChIP analysis of the binding of EZH2 (PRC2), RING1B and RYBP (PRC1) onto KSHV promoters at 4, 24, and 72 hpi and during latency. (**B**) Genome-wide recruitment of EZH2 and RING1B to the KSHV genome at 4 and 72 hpi. The Pearson correlation between the binding of EZH2 and RING1B is 0.6 at 72 hpi. Labels are the same as in Figure 3.

**Figure 6:**
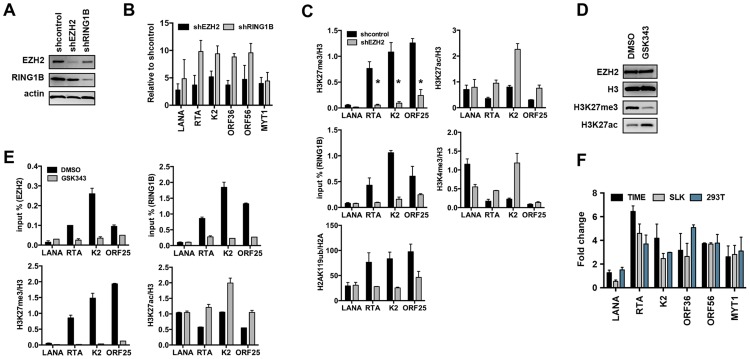
Both PRC2 and PRC1 are involved in the inhibition of lytic gene expression following *de novo* infection. (**A**) Immunoblot analysis of EZH2 and RING1B expression in shRNA-treated SLK cells. (**B**) RT-qPCR analysis of viral gene expression of KSHV infected cells upon depletion of EZH2 and RING1B expression. The expression of viral genes and MYT1 cellular gene is shown relative to that of KSHV infected SLK cells treated with scrambled shRNA. (**C**) ChIP assays show that depletion of the EZH2 expression during *de novo* infection altered the deposition profiles of histone modifications and led to the reduction of RING1B recruitment on the KSHV genome. The asterisk denotes p<0.02. (**D**) Following 2 days of treatment with either DMSO or the EZH2 inhibitor, GSK343, SLK cell lysates were used for immunoblot analysis with the indicated antibodies. (**E**) ChIP analysis of the recruitment of PcG proteins and the deposition of histone modifications on KSHV promoters in KSHV infected cells pretreated with GSK343. (**F**) GSK343-treated TIME, SLK and 293T cells were infected by KSHV for 72 hours and RT-PCR was performed to test the expression of viral genes and the cellular gene MYT1. The fold change represents the induction of viral gene expression in GSK343-treated cells compared to DMSO-treated cells.

The deposition of both repressive histone modifications, H3K27me3 and H2AK119ub, on the KSHV genome during *de novo* infection indicates that both PRC2 and PRC1 are recruited onto the viral episome. To test the binding of the PRC complexes on the viral genome, we performed ChIPs for the PRC2 subunit, EZH2, and the PRC1 subunits, RING1B and RYBP, in infected SLK cells at 4, 24, 72 hpi and again used latently-infected SLK cells as a reference. The results showed that each PcG subunit was readily detected on the promoters of the lytic genes (RTA, K2 and ORF25) at 72 hpi and during latency, while they were not recruited to the LANA promoter at any of the time points (Figure 5A). In order to demonstrate the genome-wide binding of the PRC2 and PRC1 complexes, we performed ChIP-on-chip for the EZH2 and RING1B PRC subunits in SLK cells at 4 and 72 hpi (Figures 5B and S4). This showed that EZH2 and RING1B were barely detected on the KSHV genome at 4 hpi, while they were highly enriched on the viral genome with an extensive co-occupancy at 72 hpi (Pearson correlation 0.6). To further address whether they contributed to the inhibition of lytic replication following *de novo* infection, we measured lytic gene expressions upon the shRNA-mediated depletion of either EZH2 or RING1B in SLK cells at 72 hpi. Immunoblot analysis indicated that the gene-specific shRNA treatments robustly reduced endogenous EZH2 and RING1B levels (Figure 6A). RT-PCR analysis showed that shRNA-mediated depletion of EZH2 or RING1B resulted in the induction of viral gene expression similarly to that of the PRC cellular target gene, MYT1 (Figure 6B).

Because a functional PRC2 complex has been shown to be required for the recruitment of PRC1 complex to the majority of PcG target genes, we tested whether the recruitment of PRC1 to the KSHV genome also depended on the PRC2 function. shEZH2-treated SLK cells were infected with KSHV and subjected to ChIP assays at 72 hpi (Figure 6C). shRNA-mediated depletion of the EZH2 led to the significant decline of H3K27me3 on lytic promoters, which resulted in the reduction of RING1B recruitment and H2AK119ub deposition (Figure 6C). In contrast, the levels of activating histone marks, H3K27ac and H3K4me3, were increased upon these conditions (Figure 6C). Furthermore, treatment by GSK343, a novel and specific EZH2 inhibitor [24], efficiently reduced the H3K27me3 levels without affecting the expression of EZH2 and also increased the H3K27ac levels (Figure 6D). ChIP assays also showed that GSK343 treatment resulted in the reduction of EZH2 recruitment and H3K27me3 levels on KSHV lytic promoter regions, which was accompanied by the decrease of RING1B recruitment and the increase of the activating histone mark H3K27ac (Figure 6E). Consequently, the GSK343-mediated inhibition of EZH2 increased the expression of KSHV lytic genes following *de novo* infection of various cell types (Figures 6F and S5A). Finally, we examined the effect of GSK343 treatment on *de novo* infection of 293T cells with rKSHV.219, a recombinant virus that expresses the green fluorescent protein (GFP) from the cellular EF-1α promoter and the red fluorescent protein (RFP) during lytic replication from the viral early PAN promoter [25]. This showed that GSK343 treatment resulted in significant increase of RFP expression compared to control cells (Figure S5B and C). These data collectively show that both PRC2 and PRC1 complexes bind to the KSHV genome and mediate the inhibition of lytic gene expression following *de novo* infection.

### Transcriptionally permissive chromatin assembly on the KSHV genome following *de novo* infection of oral epithelial cells

**Figure 7:**
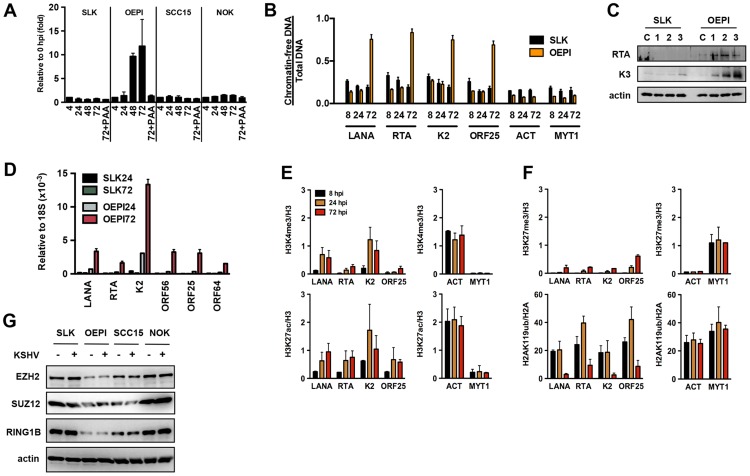
Euchromatinization of the KSHV genome in gingival oral epithelial cells following *de novo* infection. (**A**) Measurement of viral DNA replication in SLK, OEPI, SCC15 and NOK cells infected by KSHV for 4, 24, 48 and 72 hours. The viral DNA polymerase inhibitor, PAA, was applied to block the replication of KSHV. (**B**) FAIRE assay showing the degree of chromatinization of the indicated viral and cellular promoters in SLK and OEPI cells infected for 8, 24 or 72 hours. (**C**) SLK and OEPI cells were infected with KSHV for 1, 2 and 3 days and immunoblots were performed to test the expression of RTA and K3 viral proteins. Actin served as a loading control. The “C” indicates immunoblot analysis of uninfected cells. (**D**) Quantitative RT-PCR analysis of viral gene expression in KSHV infected SLK and OPEI cells. (**E**) and (**F**) ChIP analysis of the indicated histone modifications on a selection of KSHV promoters in OEPI cells at 8, 24 and 72 hpi. The cellular promoters (ACT and MYT1) were used as controls. (**G**) Comparative immunoblot analysis of the indicated cellular proteins between SLK and oral epithelial cells.

While KSHV results in latent infection in most cell types, oral epithelial cells have been reported to support lytic replication following *de novo* infection [26]. To test whether the KSHV genome undergoes a distinct chromatinization in oral epithelial cells compared to other cell types, we used three different oral epithelial cells for KSHV infection: OEPI E6/E7-immortalized human gingiva-derived epithelial cells, SCC15 human tongue squamous carcinoma cells and primary normal oral keratinocytes (NOK) cells. The susceptibility of these oral epithelial cell lines to KSHV infection was comparable with that of SLK cells based on GFP-positivity, analyzed using immunofluorescence and FACS analyses (Figure S6A and data not shown). When SLK, OEPI, SCC15 and NOK cells were infected with KSHV for 4, 24, 48 and 72 hours and measured for the viral DNA loads at each time point, KSHV replication was only detected in OEPI-infected cells and this increase in viral DNA load was sensitive to inhibition by the viral DNA polymerase inhibitor phosphonoacetic acid (PAA) (Figure 7A). Accordingly, the FAIRE assay showed that while KSHV was initially chromatinized in infected OEPI cells at 8 and 24 hpi, the viral DNA became chromatin-depleted during replication (Figure 7B). The induction of KSHV gene expression in infected OEPI cells was confirmed by immunoblotting for the lytic KSHV proteins, RTA and K3 (Figure 7C), by quantitative RT-PCR for several other lytic genes (Figure 7D), and by immunostaining for K3 expression (Figure S6). ChIP analysis of infected OEPI cells revealed increasing euchromatinization (H3K4me3 and H3K27ac) on the representative latent (LANA), IE (RTA), E (K2) and L (ORF25) promoter regions, but a lack of efficient deposition of heterochromatin histone marks (H3K27me3 and H2AK119ub) on these regions (Figures 7E and F). While OEPI, SCC15 and NOK cells were efficiently infected by KSHV (Figure S6A), the expression of Polycomb proteins was lower in OEPI cells than in NOK and SCC15 cells (Figure 7G), and the higher level of KSHV replication was detected only in OEPI cells. This indicates that the weak expression of PcG proteins correlates with the high level of KSHV replication in OEPI cells. This suggests that the weak expression of the EZH2, SUZ12 and RING1B PRC subunits may result in inadequate deposition of H3K27me3 and H2AK119ub on the lytic promoters of infected OEPI cells, resulting in the activation of lytic gene expression. It should be noted that despite the viral DNA replication in OEPI cells following *de novo* infection, we did not detect infectious virus particles, suggesting that additional factors downstream of viral DNA replication may prevent the production of infectious virions.

Taken together, these data suggest that the differential epigenetic modification of the KSHV genome in distinct cell types may determine whether KSHV establishes latent or lytic gene expression program following *de novo* infection.

## Discussion

**Figure 8:**
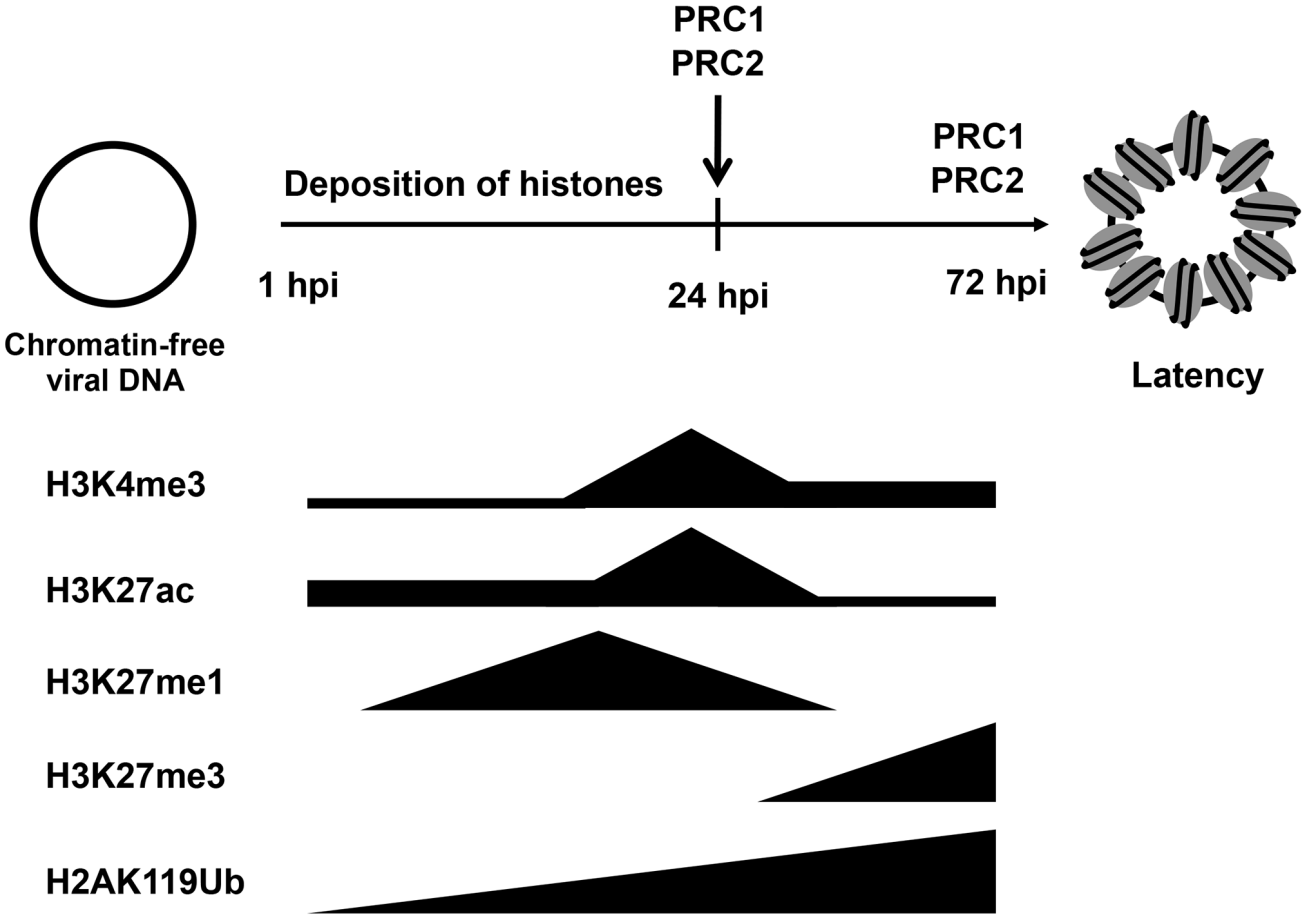
Biphasic euchromatin-to-heterochromatin transition on the KSHV genome following *de novo* infection. As soon as the viral genome enters the nucleus, histones are recruited on the viral DNA, resulting in the chromatinization of the KSHV genome. The viral genome initially adopts a transcriptionally permissive chromatin, characterized by high levels of the H3K27ac and H3K4me3 and this is accompanied by the transient expression of lytic genes. Subsequently, the PRC2 and PRC1 are recruited to viral genome, where they are responsible for the enrichment of H3K27me3 and H2Ak119ub on the viral chromatin as well as the repression of lytic genes. During latency, both PRC2 and PRC1 remain on the KSHV genome for the maintenance of the repression of lytic genes.

While the KSHV genome is histone-free in the virions, the viral DNA adopts a highly organized chromatin structure in latently infected cells, which is an essential step in establishing the latency-associated viral gene expression program necessary for persistent infection of the host [10]. A previous study by Gunther and Grundhoff has shown that at 5 days after infection of SLK cells, KSHV adopts a highly structured chromatinized episome that resembles the viral chromatin structure found in latently infected B cell lymphoma cells [12]. Here, we demonstrate that the latent chromatin structure of KSHV gradually develops following *de novo* infection and is fully established as early as 3 days post-infection. Based on our results, we propose that the KSHV genome undergoes a spatially and temporally ordered chromatinization following *de novo* infection prior to the establishment of latency (Figure 8). We demonstrated that the viral DNA rapidly associates with histones after infection and that initially, there is a transient enrichment of H3K4me3 and H3K27ac activating histone marks on the viral chromatin and concomitant expression of lytic genes. This is followed by the decline of activating histone marks and the transition from a transcriptionally active viral chromatin to a H3K27me3/H2AK119ub-enriched heterochromatin, a transition that is regulated by the PRC2 and PRC1 cellular transcription repressor complexes and ultimately results in the inhibition of lytic gene expression and the establishment of latency (Figure 8). Thus, our results indicate that the KSHV genome undergoes a biphasic chromatinization after *de novo* infection prior to the establishment of latency.

Our previous study has shown that the distinct chromatin domains of the latent KSHV genome are characterized by different combinations of histone modifications [10], [11], [12]. The results of this study show that the KSHV genome undergoes a specific biphasic euchromatin-heterochromatin transition upon *de novo* infection. Both studies indicate that activating and repressive histone marks are enriched only on specific viral genomic regions in the early stage of infection and during latency. For example, the deposition of H3K27me3 is always excluded from the latency-associated locus but enriched on lytic genes-encoding regions throughout both *de novo* infection and latency (Figures 2 and 3). Also, the deposition of H3K4me3 occurs mainly on latent genes and some IE/E genes at 4 hpi and during latency. These findings indicate targeted recruitment of specific histone-modifying enzyme complexes to different sites of the KSHV genome from the beginning of infection, a process that is likely orchestrated by sequence-specific DNA-binding factors. These factors might be, for example, the CTCF/cohesin complex, which is involved in the regulation of nuclear organization of the KSHV chromosome or RBP-Jκ, which controls viral transcription by binding to many KSHV promoters [27], [28]. Additional studies will be required to demonstrate how the positions of these chromatin domains are specifically determined on the KSHV genome and what types of DNA-binding factors are involved in the recruitment of chromatin regulatory complexes onto the KSHV genome.

Our data also show that the deposition of activating and repressive histone marks on the KSHV genome occurs in a temporally ordered manner following *de novo* infection. One of the striking examples of this is the differential modification of H3K27 (Figure 2). In mammalian cells, H3K27 can either be acetylated by the histone acetyltransferases CBP/p300 or mono-, di-, or trimethylated by EZH2 [29]. By using sequential ChIP assays, we found that the acetylation of H3K27 is followed by its gradual switch to methylation on the same KSHV genome, indicating the sequential action of different histone modifying enzymes on the same viral genome following *de novo* infection. We also observed that the decrease of H3K27ac on lytic promoters resulted in increasing levels of H3K27me3 in a coordinated manner, further supporting that the histone modifying enzymes are recruited to the KSHV genome in a temporally ordered manner following *de novo* infection.

Because the KSHV genome is initially chromatin-free following infection, the viral promoters are easily accessible by the RNAPII transcription machinery to induce viral transcription, which might explain the transient expression of lytic genes during *de novo* infection (Figure 1) [17]. RNAPII has been shown to interact with H3K4me3 histone methyltransferases and the H3K27me3 demethylase JMJD3 [30], [31], [32], [33]. Therefore, these histone modifying enzymes might be recruited onto lytic promoters by RNAPII and thereby contribute to the deposition of histone modifications on the viral chromatin during *de novo* infection. In addition, viral proteins expressed during *de novo* infection can also be involved in the modulation of the evolving viral chromatin structure. Indeed, we found that the transient expression of the lytic protein RTA had a significant effect on the deposition of activating histone modifications on lytic promoters. In the absence of RTA, the level of both H3K4me3 and H3K27ac was significantly lower on the KSHV genome after infection and this was accompanied by the reduced expression of several lytic genes (Figure 4). RTA binding to lytic promoters also recruited CBP, the histone acetyltransferase of H3K27ac to lytic promoters during *de novo* infection. These data suggest that the RNAPII transcription machinery and the viral transcription factor RTA may promote the deposition of activating histone marks on the KSHV genome during *de novo* infection, contributing to the temporal induction of lytic genes.

RTA is a potent viral transcription factor, which is sufficient for the induction of the lytic gene expression program in infected cells [34]. However, upon *de novo* infection, KSHV usually does not undergo lytic replication, even though RTA is expressed. This might be due to the weak endogenous promoter of RTA, which seems to be prone to repression and thus, it is unable to sustain its continuous expression after infection. Indeed, heterochromatin-associated cellular factors such as the PRC2 complex, HDACs and transcription repressor KAP-1 have been shown to be recruited onto the RTA promoter, leading to the silencing of the promoter [8], [11], [35]. On the other hand, KSHV infection of RTA-expressing cells (Figure S3) or infection of cells with a recombinant KSHV constitutively expressing RTA [36] resulted in efficient lytic replication. Thus, these data indicate that the temporal expression of RTA during *de novo* infection may not be sufficient to induce the full cycle of KSHV lytic replication, thereby leading to the establishment of latency.

We have previously shown that PRC2 is involved in the repression of lytic genes during latency [11], [12]. Here, we have found that the PRC1 complex is also recruited onto the KSHV genome and both PRCs are involved in the repression of lytic genes following *de novo* infection (Figures 5 and 6). Furthermore, while PRC1 was preferentially recruited onto the lytic gene promoters via H3K27me3 pre-deposited by PRC2, PRC1 may also bind to specific lytic gene regions independently of PRC2 via its RYBP DNA-binding factor [15]. We found that, during *de novo* infection, the binding of RYBP on the viral genome precedes that of EZH2, indicating a sequential recruitment of PcG proteins, where RYBP may recruit PRC1 independently of PRC2 at specific lytic promoters (Figure 5). These PRCs have been shown to be recruited to their cellular target loci by various non-coding RNAs and distinct transcription factors [13]. Thus, it would be intriguing to determine what host or viral factors are responsible for the recruitment of PRCs on the KSHV episome during *de novo* infection and latency.

Surprisingly, while KSHV infection results in latency in most cell types, we found that in infected human OEPI cells, the KSHV genome acquires H3K4me3/H3K27ac-enriched chromatin, which is accompanied by lytic replication (Figure 7). Interestingly, the expression of both EZH2 and RING1B was lower in OEPI cells compared to SLK and NOK cells, likely contributing to the reduced level of H3K27me3 and H2AK119ub on the KSHV genome in OEPI cells. This suggests that the levels of chromatin regulatory factors that KSHV encounters during infection of host cells may influence the outcome of viral infection. However, overexpression of EZH2 alone in OEPI cells was not sufficient to force KSHV to establish latency (unpublished results). It is possible that all or most of the components of PRC complexes must be overexpressed in a specific ratio or additional repressors, other than PRC complexes, are required for the establishment of KSHV latency.

Establishment of latency is a common feature of all herpesviruses and is characterized by the silencing of lytic genes and the inhibition of viral replication [37]. Emerging evidence shows that Polycomb proteins are involved in the repression of some lytic genes of Herpes simplex virus type 1 (HSV-1) [38], [39], Human cytomegalovirus (HCMV) [40] and Epstein-Barr virus [41], [42]. These observations indicate that PRCs may function as part of a common intrinsic immunity defense system against all herpesviruses, wherein they act as inhibitors of viral replication and gene expression. The incoming viral genomes of HSV-1 and HCMV have also been shown to rapidly associate with histones following *de novo* infection [43], [44]. As with KSHV infection of oral epithelial cells, the viral chromatin of HSV-1 and HCMV is enriched in activating histone marks, facilitating lytic replication [45]. However, the regulation of chromatinization of the HSV-1 and HCMV genomes during the establishment of latent infection has yet to be studied.

In summary, our results are the first comprehensive study showing that, following *de novo* infection of different cell types, the KSHV genome undergoes a well-orchestrated transition between distinct chromatin states and that this process is regulated by site-specific recruitment of chromatin regulatory factors onto the KSHV genome. Because of the compact size of its genome and the presence of several unique viral DNA sequences, KSHV may use a specific set of transcription and chromatin regulatory factors to regulate its viral chromatin structure. Therefore, the identification of the relevant cellular and viral proteins involved in the regulation of chromatin structure of KSHV can lead to the discovery of new therapeutic targets for controlling KSHV infection and pathogenesis.

## Materials and Methods

### Cells, viruses and *de novo* infection

293T and SLK cells were maintained in DMEM medium supplemented with 10% FBS, 100 U/ml penicillin and 100 µg/ml streptomycin (P/S). TIME cells were cultured in VasculoLife VEGF medium according to the manufacturer's specifications (Lifeline Cell Technology). The generation of the iSLK cell line is described elsewhere [18]. iSLK cells were cultured in DMEM medium supplemented with 10% FBS, P/S, 1 µg/ml puromycin and 250 µg/ml G418. The iSLK cell lines carrying BAC16 or BAC16RTAstop were maintained in the presence of 1 mg/ml hygromycin. The construction of BAC16RTAstop virus was described previously [21]. NOK (gift from Yang Chai, USC), SCC15 (ATCC) and gingival OEPI epithelial cell lines were cultured in Keratinocyte-SFM medium (Gibco) according to the manufacturer's instructions. The primary gingival epithelial cell line was a generous gift from Jennifer Webster-Cyriaque (UNC, Chapel Hill), which was immortalized by the papillomavirus E6/E7 to make OEPI. KSHV was prepared from iSLKBAC16 and iSLKBAC16RTAstop cell cultures by treatment with 1 µg/ml doxycycline and 3 mM sodium butyrate and the viral titer was calculated as described previously [21]. *De novo* infection was performed by spin-infection using a MOI of 1 (2000 rpm, 45 min at 30°C). After infection the media was changed and the infected cells were harvested at the indicated time points.

### Antibodies and inhibitors

The following antibodies were used in ChIPs and/or immunoblots: rabbit anti-histone H3 (Abcam ab1791), rabbit anti-H3K27me1 (Millipore 07-448), rabbit anti-H3K27me3 (Millipore 07-448, Active Motif 39155 and Cell Signaling 9756), rabbit anti-H3K4me3 (Millipore 04-745 and Active Motif 39159), rabbit anti-H2A (Millipore 07-146), rabbit anti-H2AK119ub (Cell Signaling 8240), rabbit anti-H3K27ac (Abcam ab4729), mouse anti-BMI1 (Millipore 05-1322), rabbit anti-CBP (sc-369), rabbit anti-RING1B (Abcam ab101273), mouse anti-EZH2 (BD Biosciences 612666), rabbit anti-SUZ12 (Abcam ab12073), rabbit anti-RYBP (Abcam ab5976), rabbit anti-Spt5 (sc-28678) and mouse anti-actin (Abcam). The rabbit anti-RTA antibody was a generous gift from Drs. Yoshihiro Izumiya and Hsing-Jien Kung (UC Davis, Sacramento). LANA and K3 KSHV protein-specific antibodies have been described previously [11]. GSK343 was obtained from Structural Genomics Consortium (Toronto, Canada). GSK343 was used at 50 µg/ml in cell culture.

### ChIP and ChIP-on-chip assays

ChIPs and ChIP-on-chips were performed as have been published previously, with a few modifications [11]. The primer sequences used in ChIP-qPCR are listed in Table S1. ChIP graphs show the average of at least two independent experiments. For ChIP-on-chip, 10 µg of chromatin and 2 µg of antibodies were used for each reaction. The ChIP-on-chips were performed with a custom-designed Agilent tiling microarray, as described previously [11]. Briefly, amplified ChIP DNA and input DNA samples (2 ug) were submitted to the UC Davis Comprehensive Cancer Center Genomics Shared Resource for target labeling (Cy3, Cy5), array processing, and microarray scanning. Raw image files were processed with Agilent Feature Extraction software (version 10.5.1.1) to quantify feature signal intensities and to perform normalization, dye bias correction, and background subtraction. The raw data was pre-processed by blank subtraction (one-step Tukey biweight subtraction) and intra-array (dye-bias) median normalization in order to equalize the ChIP (Cy5) and input (Cy3) DNA channels. Binding events, or enrichments, were represented as increases in the ratios of the ChIP to input DNA signal intensities. The ChIP-on-chip data is freely available at the GEO repository (GSE51660). Data analysis of the 1 kb genomic region surrounding the TSS of each viral gene was performed as described in details previously [11]. The ChIP-on-chip data was imported into Java TreeView (version 1.1.6r.4) for visualization.

### FAIRE assay

Formaldehyde-assisted isolation of regulatory elements (FAIRE) analysis was performed as described by Giresi et al with some modifications [46]. DNA was purified from an equal amount of formaldehyde-crosslinked and de-crosslinked chromatins, followed by the quantification of DNAs via qPCR using viral or cellular promoter specific primers. DNA purified from the formaldehyde-crosslinked chromatin represents chromatin-free DNA, while DNA derived from de-crosslinked chromatins is for the calculation of total DNA. The ratio of chromatin-free and total DNAs shows the degree of chromatinization of a given genomic DNA fragment in cells.

### RNA purification and RT-qPCR

Total RNA was extracted from cells using Tri reagent (Sigma) according to the manufacturer's instructions. 1 µg of total RNA was treated with DNase I (Sigma), reverse transcribed by iScript cDNA Synthesis kit (Bio-Rad) and the cDNA was quantified by qPCR using gene specific primers. The primer sequences are listed in Table S1. The relative level of gene expression was calculated by the 2^−dCt^ or the ddCt method, where either actin or 18S was used for normalization. The RT-qPCR graphs represent the average of at least two independent experiments.

### Lentiviral shRNA knockdown

The pLKO.1 lentiviral vector was used to express the EZH2 and RING1B specific shRNAs. Supernatants from 293T cells transfected by the shRNA and packaging vectors were collected 60 hours post-transfection, followed by concentration of the virus (24000 rpm, 1.5 hr, 4°C) and used for infection of cells in the presence of 10 µg/ml polybrene. 2 days after lenti-shRNA infection, the cells were split, and then infected with KSHV the following day.

### Immunofluorescent analysis

Cells were fixed with 4% paraformaldehyde and then permeabilized by 0.5% Triton X100. 10% FBS was used for blocking nonspecific antibody binding, followed by incubation of cells with antibodies against the KSHV protein K3. After extensive washing with PBS, TRITC-conjugated secondary antibody was applied, followed by Hoechst staining.

## Supporting Information

Figure S1 **Immunofluorescent analysis of SLK cells infected with BAC16 KSHV for 8, 24 or 72 hours at MOI of 1 and latently infected SLK cells.**
(PPTX)

Figure S2 **Analysis of the deposition of histone modifications on KSHV promoters during** ***de novo*** **infection of TIME cells.** ChIPs were performed at the indicated viral promoters with TIME cells at 8, 24 and 72 hpi and with latently infected TIME cells. Each histone modification ChIP was normalized for the amount of the relevant histones at each promoter as in Figure 2.
(PPTX)

Figure S3 **Constitutive replication of KSHV in RTA-expressing SLK cells.** (**A**) Measurement of KSHV DNA replication in Dox uninduced (−) and induced (+) iSLK cells at 24 and 72 hpi. (**B**) Immunoblot analysis of KSHV protein expression in Dox-untreated or Dox-treated iSLK cells. (**C**) RT-qPCR test of KSHV gene expression in Dox-untreated or Dox-treated iSLK cells. (**D**) Recruitment of histones H3 and H2A on KSHV promoters in Dox-untreated or Dox-treated iSLK cells at 72 hpi.
(PPTX)

Figure S4 **Recruitment of EZH2 and RING1B to the gene regulatory regions of KSHV genes at 72 hpi.**
(PPTX)

Figure S5 (**A**) TIME, SLK or 293T cells were treated with GSK343 in the absence or presence of KSHV infection and cell viability was checked by cell counting every 2 days. (**B**) Naïve 293T cells were pre-treated with GSK343 or DMSO for 48 hours and then infected with rKSHV.219 for an additional 72 hours. GFP and RFP expression was analyzed by fluorescent microscopy. (**C**) Quantification of RFP positive cells, p<0.0001.
(PPTX)

Figure S6 (**A**) Infection of SLK and oral epithelial cells OEPI, SCC15 and NOK with BAC16 KSHV. Photos were taken at 24 hpi. (**B**) Detection of the expression of the KSHV lytic protein K3 in KSHV infected OEPI cells at 72 hpi using immunofluorescent analysis.
(PPTX)

Table S1 **Primer sequences used in this study.**
(DOCX)

Table S2 **Pearson correlations in ChIP-on-chip.**
(DOCX)
