# Supplementary figures and images for: Biphasic Euchromatin-to-Heterochromatin Transition on the KSHV Genome Following De Novo Infection
Source: PLoS Pathog. 2013 Dec 19;9(12):e1003813. doi: 10.1371/journal.ppat.1003813 (PMC3868514; doi:10.1371/journal.ppat.1003813)

## Slide 1
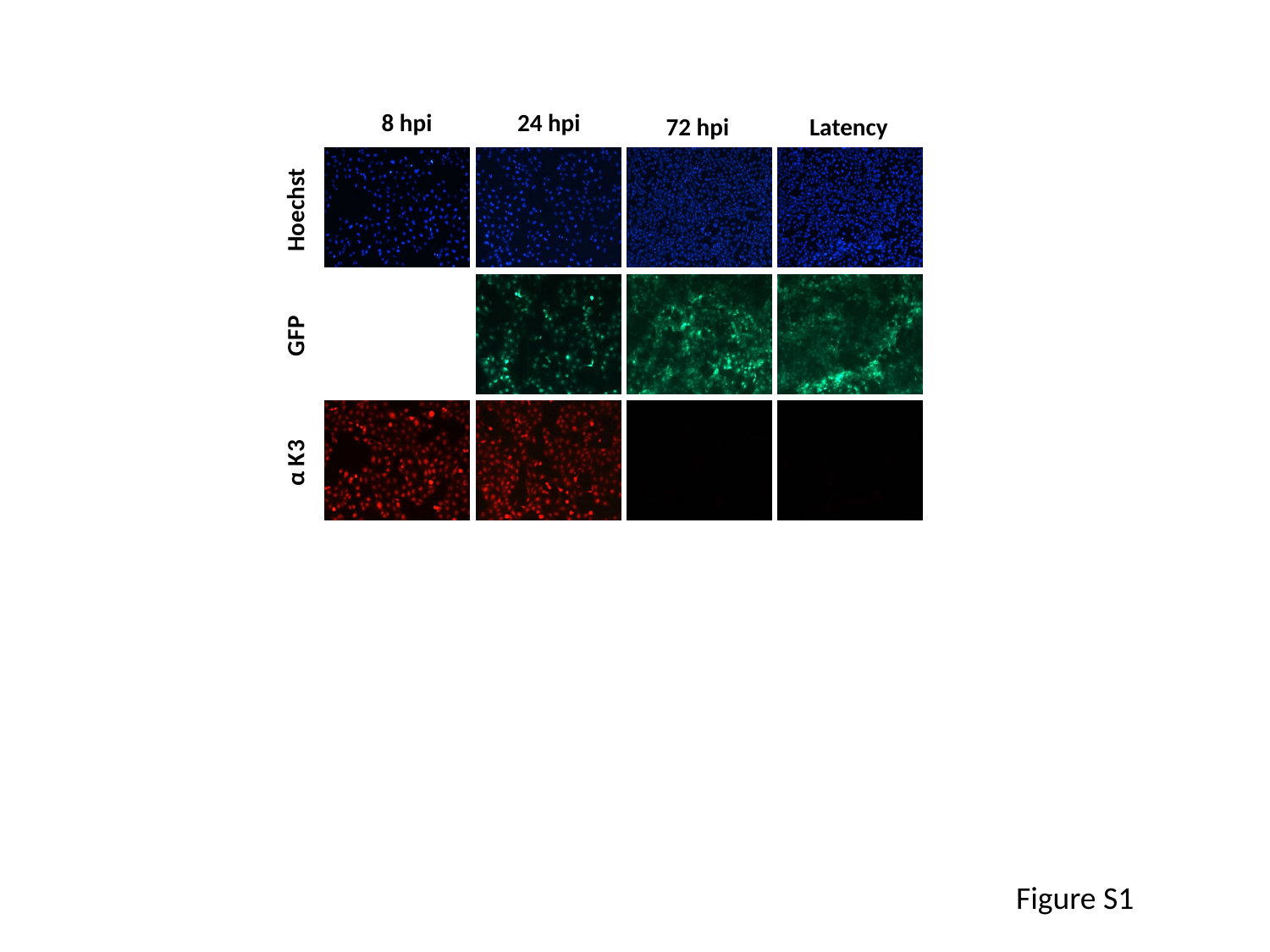

8 hpi
24 hpi
72 hpi
Latency
Hoechst
GFP
α K3
Figure S1

Supplement: Figure S1 — Immunofluorescent analysis of SLK cells infected with BAC16 KSHV for 8, 24 or 72 hours at MOI of 1 and latently infected SLK cells. (PPTX) [file ppat.1003813.s001.pptx]

## Slide 1
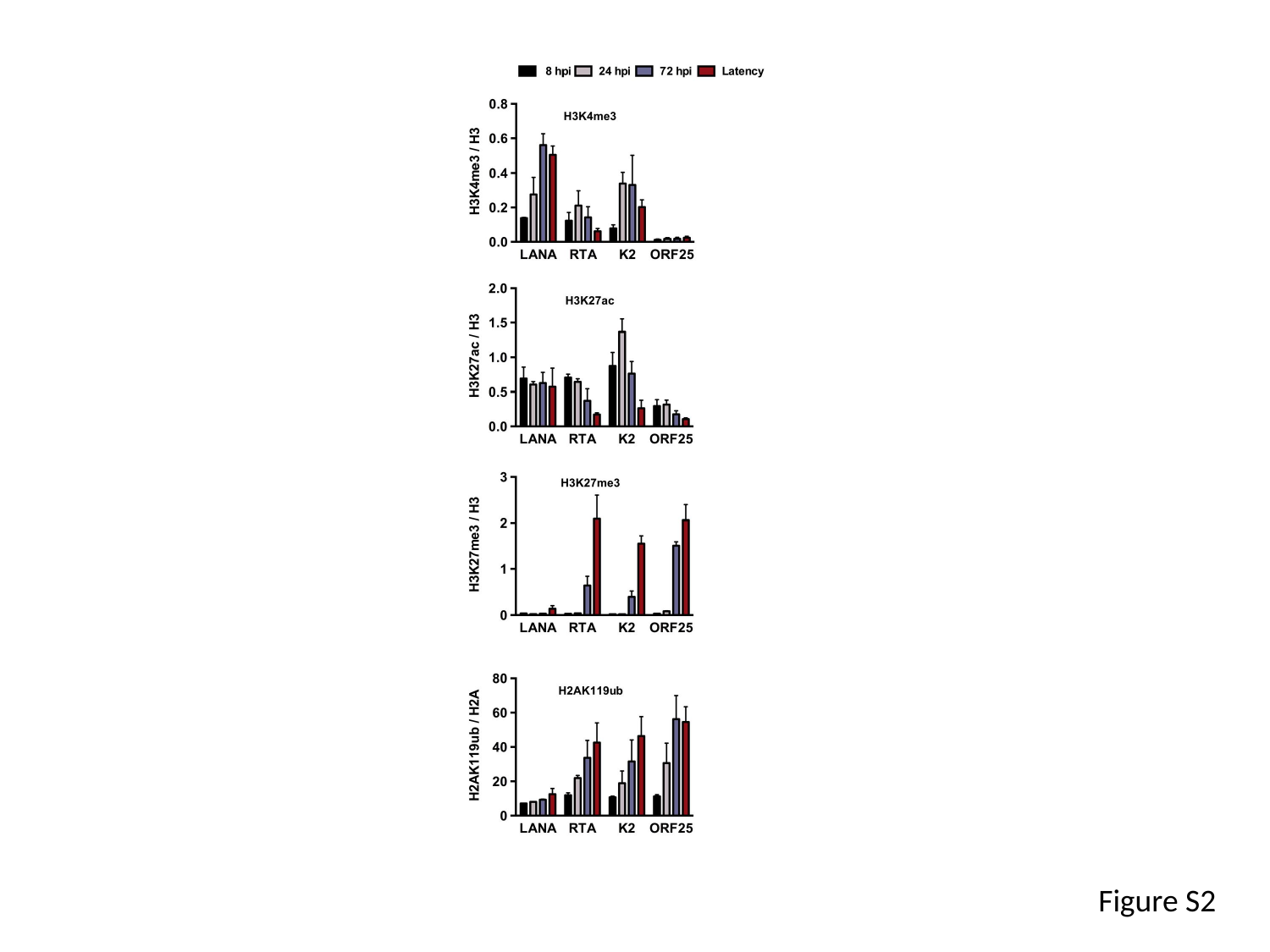

Figure S2

Supplement: Figure S2 — Analysis of the deposition of histone modifications on KSHV promoters during de novo infection of TIME cells. ChIPs were performed at the indicated viral promoters with TIME cells at 8, 24 and 72 hpi and with latently infected TIME cells. Each histone modification ChIP was normalized for the amount of the relevant histones at each promoter as in Figure 2. (PPTX) [file ppat.1003813.s002.pptx]

## Slide 1
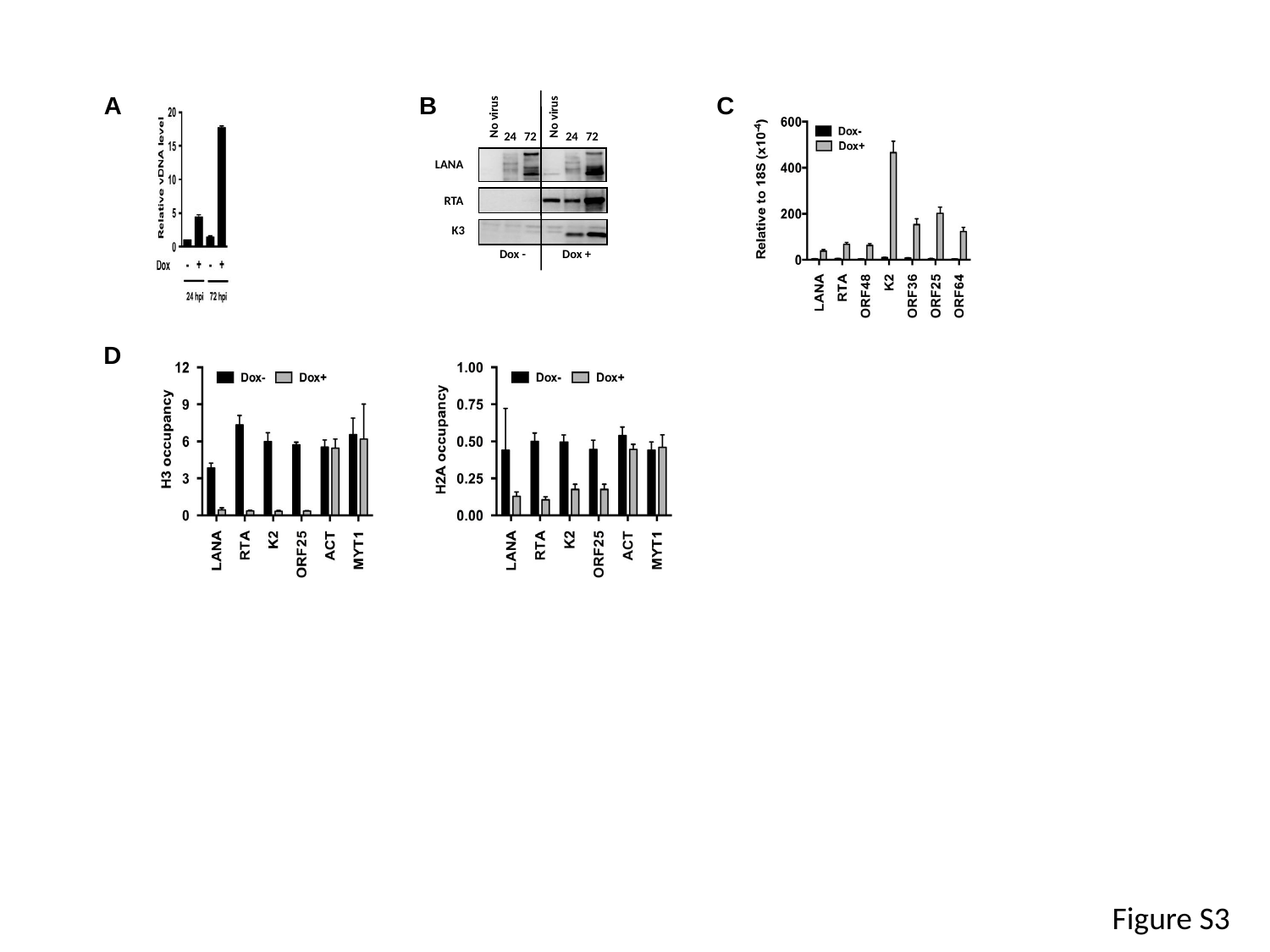

No virus
No virus
24
72
24
72
LANA
RTA
K3
Dox -
Dox +
A
B
C
D
Figure S3

Supplement: Figure S3 — Constitutive replication of KSHV in RTA-expressing SLK cells. (A) Measurement of KSHV DNA replication in Dox uninduced (−) and induced (+) iSLK cells at 24 and 72 hpi. (B) Immunoblot analysis of KSHV protein expression in Dox-untreated or Dox-treated iSLK cells. (C) RT-qPCR test of KSHV gene expression in Dox-untreated or Dox-treated iSLK cells. (D) Recruitment of histones H3 and H2A on KSHV promoters in Dox-untreated or Dox-treated iSLK cells at 72 hpi. (PPTX) [file ppat.1003813.s003.pptx]

## Slide 1
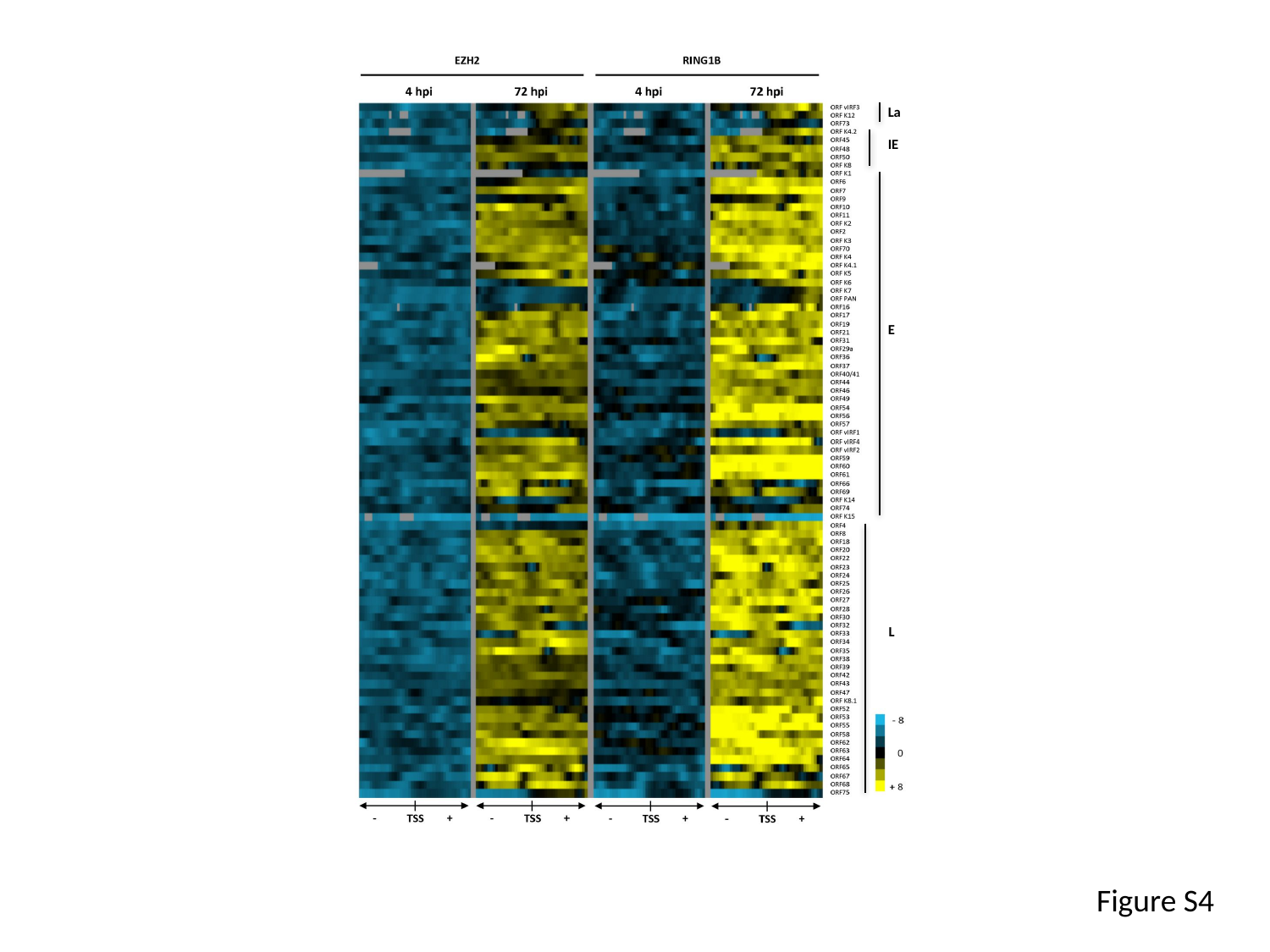

La
IE
E
L
Figure S4

Supplement: Figure S4 — Recruitment of EZH2 and RING1B to the gene regulatory regions of KSHV genes at 72 hpi. (PPTX) [file ppat.1003813.s004.pptx]

## Slide 1
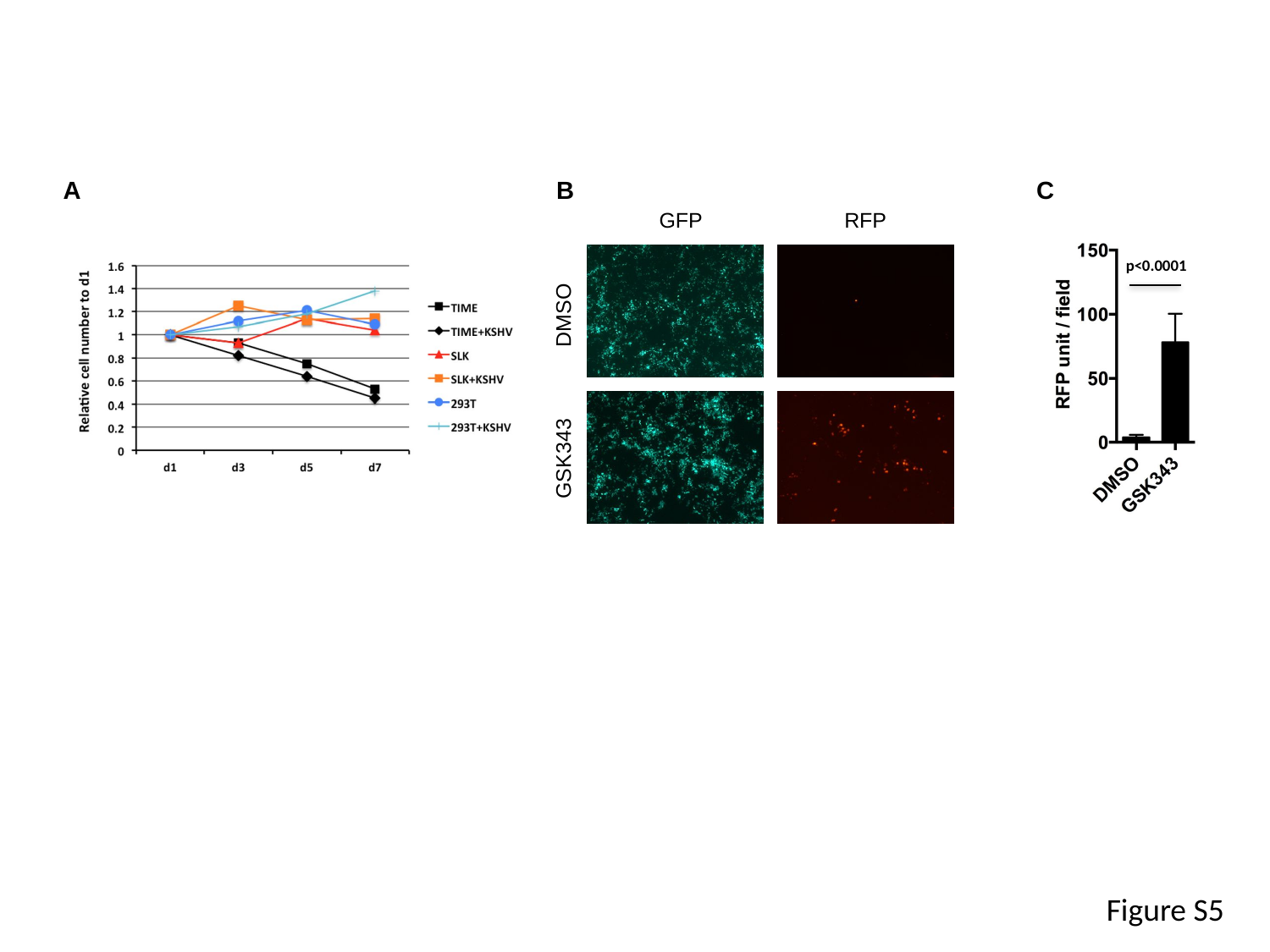

A
B
C
GFP
RFP
p<0.0001
DMSO
GSK343
Figure S5

Supplement: Figure S5 — (A) TIME, SLK or 293T cells were treated with GSK343 in the absence or presence of KSHV infection and cell viability was checked by cell counting every 2 days. (B) Naïve 293T cells were pre-treated with GSK343 or DMSO for 48 hours and then infected with rKSHV.219 for an additional 72 hours. GFP and RFP expression was analyzed by fluorescent microscopy. (C) Quantification of RFP positive cells, p<0.0001. (PPTX) [file ppat.1003813.s005.pptx]

## Slide 1
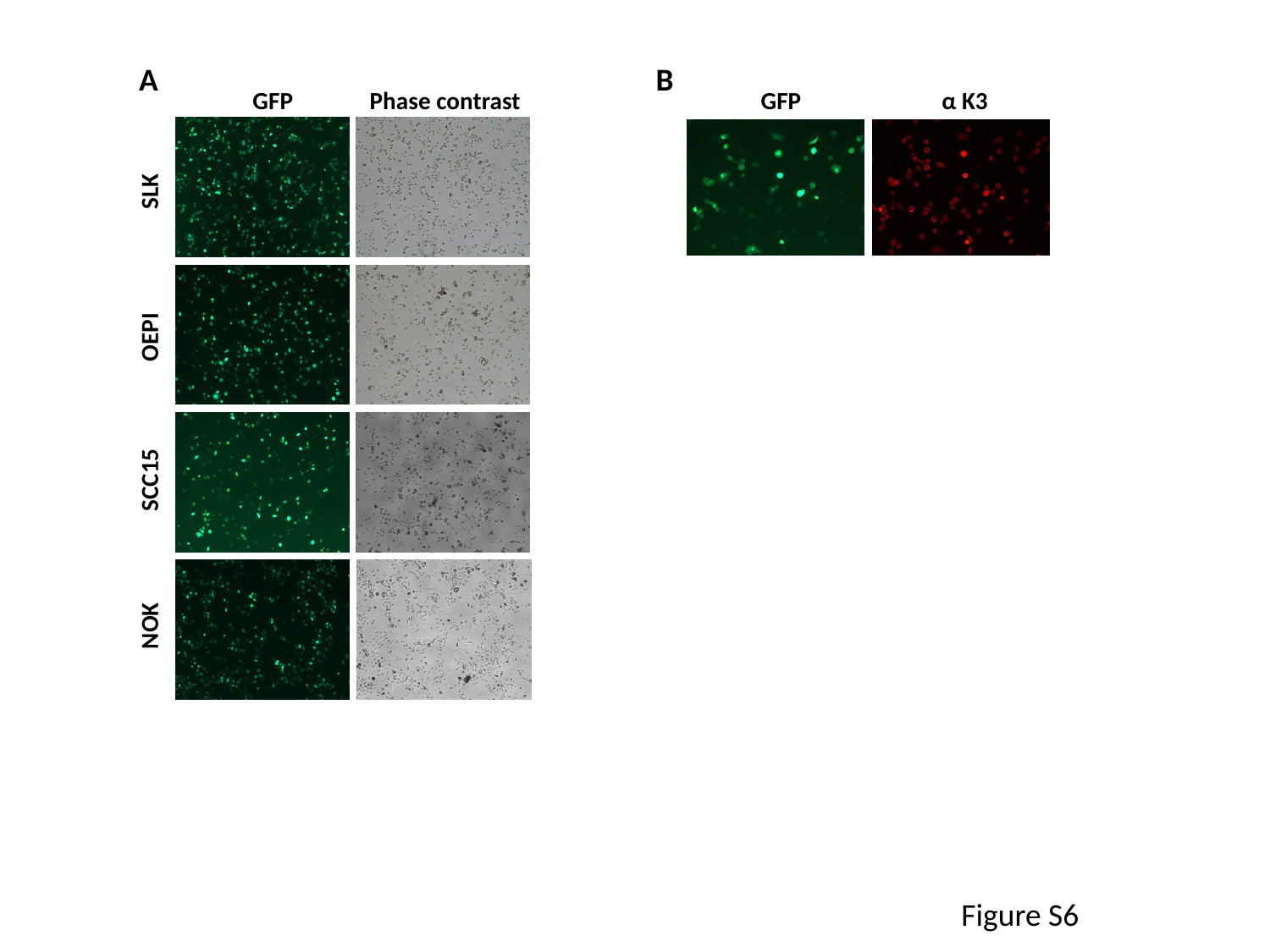

A
B
GFP
Phase contrast
GFP
α K3
SLK
OEPI
SCC15
NOK
Figure S6

Supplement: Figure S6 — (A) Infection of SLK and oral epithelial cells OEPI, SCC15 and NOK with BAC16 KSHV. Photos were taken at 24 hpi. (B) Detection of the expression of the KSHV lytic protein K3 in KSHV infected OEPI cells at 72 hpi using immunofluorescent analysis. (PPTX) [file ppat.1003813.s006.pptx]
