# Supplementary material for: Biphasic Euchromatin-to-Heterochromatin Transition on the KSHV Genome Following De Novo Infection
Source: PLoS Pathog. 2013 Dec 19;9(12):e1003813. doi: 10.1371/journal.ppat.1003813 (PMC3868514; doi:10.1371/journal.ppat.1003813)
Supplement: Table S1 — Primer sequences used in this study. (DOCX) [file ppat.1003813.s007.docx]

| **Promoter primers for ChIP-qPCR** | |  |
| --- | --- | --- |
|  |  |  |
|  | **Forward primer** | **Reverse primer** |
| **LANA** | GTTTATAAGTCAGCCGGACCAA | GATATAACTCCGCCCTCCACTA |
| **RTA** | AAGACACTGACCCACCAAGG | GGTGCCACCAATGTATGACC |
| **K2** | CATACGCAGCCAAGCTATCA | GCTAGCACAGCAAATTGAGA |
| **ORF25** | AGTTGTCGGTGTCTATCTGT | TGCAGAGCGATACGCAGACT |
| **ORF10** | CTCTTGCGCTATGTGGGACAA | GGCAGTTAGACACCGTTATCT |
| **K3** | CTAGAGATAGTGAGCCAGGT | CTGGTACGGTCACTTGTTGCA |
| **ORF70** | AGTTACCTCATGCTGAATCGA | ACAAACTTCCAGGATCTATCA |
| **ORF56** | CGTTCTGAATAGACTGCAGT | CAAGATCACAGACTACCTGA |
| **ORF46** | GATCGGCGAAGTGGATAGAGT | CCCTATTGGTCACATCTCACG |
| **ORF47** | CCCCAACACAAGGACCTTTA | GCTTTTGGATACCCTGGTGA |
| **ACT** | CCACAGCCAGAGGTCCTCAG | AGGAGCTCTTGGAGGGCATG |
| **MYT1** | GATTCTGTATTCATCATGGA | GCTGTGAGTGCTAGGATGTCT |
|  |  |  |
| **Primers for RT-qPCR** | |  |
|  |  |  |
| **LANA** | GAGTCTGGTGACGACTTGGAG | AGGAAGGCCAGACTCTTCAAC |
| **RTA** | TTGCCAAGTTTGTACAACTGCT | ACCTTGCAAAGACCATTCAGAT |
| **ORF45** | CCATACAGCGACCCTGATGA | CCGATTCTCTGACTCAATACT |
| **ORF48** | CCACATCTTCATAGAGCACAT | ATTGCATCACCAGGGTATCCA |
| **K2** | TCACTGCGGGTTAATAGGATTT | CATGACGTCCACGTTTATCACT |
| **ORF36** | ATTGCCAACGACCTGATGCA | ACTCCAGTCCAGCTGCAGCA |
| **ORF56** | CACAGATTCCCGTCAATACAAA | GTATCTTCAGTAGGCGGCAGAG |
| **ORF57** | AGGGATATCACCGCTCTCATAAGA | CTGCGGTTTCTCGACGGCAACTCA |
| **K6** | ATGCTGCGTTAGCGTACTGCT | GAACCCGTAGCAGCAGCTAT |
| **K7** | TGCCGCTTCACCTATGGATT | ACGCAATCAACCCACAATCG |
| **ORF46** | GGTACGGGTCCTGGCCTAAGATCA | ACTGCTCTCGCTGCTCAGACAGAT |
| **ORF25** | ACAGTTTATGGCACGCATAGTG | GGTTCTCTGAATCTCGTCGTGT |
| **vIRF2** | TCATGGCTGGTTCCTGCGTCAAGT | AGGACCGCCAATCGAGCCAGACAG |
| **MYT1** | CACTGTGCGGAAGAGTTACT | CTCATGCATGGCTAAGATCT |
| **18S** | TTCGAACGTCTGCCCTATCAA | GATGTGGTAGCCGTTTCTCAGG |
