# Supplementary material for: Biphasic Euchromatin-to-Heterochromatin Transition on the KSHV Genome Following De Novo Infection
Source: PLoS Pathog. 2013 Dec 19;9(12):e1003813. doi: 10.1371/journal.ppat.1003813 (PMC3868514; doi:10.1371/journal.ppat.1003813)
Supplement: Table S2 — Pearson correlations in ChIP-on-chip. (DOCX) [file ppat.1003813.s008.docx]

|  | **H3K27Ac_4hpi** | **H3K27Ac_24hpi** | **H3K27Ac_72hpi** | **H3K4m3_4hpi** | **H3K4m3_24hpi** | **H3K4m3_72hpi** | **H3K27m3_24hpi** | **H3K27m3_72hpi** |
| --- | --- | --- | --- | --- | --- | --- | --- | --- |
| **H3K27Ac_4hpi** | 1.00 |  |  |  |  |  |  |  |
| **H3K27Ac_24hpi** | 0.56 | 1.00 |  |  |  |  |  |  |
| **H3K27Ac_72hpi** | 0.44 | 0.82 | 1.00 |  |  |  |  |  |
| **H3K4m3_4hpi** | 0.64 | 0.63 | 0.51 | 1.00 |  |  |  |  |
| **H3K4m3_24hpi** | 0.59 | 0.66 | 0.46 | 0.63 | 1.00 |  |  |  |
| **H3K4m3_72hpi** | 0.50 | 0.70 | 0.63 | 0.68 | 0.83 | 1.00 |  |  |
| **H3K27m3_24hpi** | 0.09 | 0.11 | -0.07 | -0.05 | 0.07 | -0.07 | 1.00 |  |
| **H3K27m3_72hpi** | 0.15 | 0.14 | -0.08 | -0.04 | 0.16 | -0.08 | **0.81** | 1.00 |
